# Comparison of the Health Implications on the Use of As and Cd Contaminated Water Supply between Urban and Rural Communities

**DOI:** 10.1155/2014/797603

**Published:** 2014-11-04

**Authors:** H. Zailina, H. Najibah, A. Nadia Aiezzati, S. M. Praveena, I. Patimah

**Affiliations:** ^1^Department of Environmental and Occupational Health, Faculty of Medicine and Health Sciences, UPM, 43400 Serdang, Selangor, Malaysia; ^2^Department of Biomedical Science, Faculty of Medicine and Health Sciences, UPM, 43400 Serdang, Selangor, Malaysia

## Abstract

A cross-sectional study was carried out to determine the arsenic (As) and cadmium (Cd) concentrations in blood, urine, and drinking water as well as the health implications on 100 residents in an urban and a rural community. Results showed the blood As, urinary Cd, DNA damage, and water As and Cs were significantly (*P* < 0.001) higher in the rural community. Findings showed significant (*P* < 0.005) correlations between blood As and DNA damage with household income, years of residence, and total glasses of daily water consumption among the rural residents. The urinary NAG concentrations, years of residence, milk powder intake (glass/week), and seafood intake (per week) were significantly correlated (*P* < 0.005) with urinary Cd concentrations among respondents. In addition, urinary Cd level significantly influenced the urinary NAG concentrations (*P* < 0.001). The rural respondents experienced significantly higher lymphocyte DNA damage and blood As influenced by their years of residence and water consumption. The Cd in drinking water also resulted in the rural respondents having significantly higher urinary NAG which had a significant relationship with urinary Cd.

## 1. Introduction

Arsenic (As) is a class 1 human carcinogen [1]. Deoxyribonucleic acid (DNA) damage is the abnormalities of the DNA sequence in cells. Normally, DNA damages can be recognized by enzymes, and thus it can be correctly repaired if the undamaged sequence in the complementary DNA strand or in a homologous chromosome is available for copying. The DNA damages are a special problem in nondividing or slowly dividing cells, where unrepaired damages will tend to accumulate over time. On the other hand, in rapidly dividing cells, unrepaired DNA damages that do not kill the cell by blocking replication will tend to cause replication errors and thus mutation. These mutant cells can give rise to cancer. Urinary cadmium (Cd) is one of the biomarkers that indicates long-term exposure of Cd that can be related to urinary N-acetyl-*β*-D-glucosaminidase (NAG). NAG, known as the renal biomarker, detects renal dysfunction especially on proximal tubules dysfunction. NAG is a high molecular-weight lysosomal enzyme found in many tissues of the body. NAG is an enzyme derived from the kidney and found in the urine of normal healthy person. It is a sensitive and early marker of renal tubular dysfunction [2].

Arsenic is mainly absorbed by both oral and inhalation routes. Environmental exposure of As is through ingestion [3]. Toxicity of As involves reduction or oxidation reactions that interconvert As(III) and As(V) and methylation reactions. Human body has the ability to change inorganic As to organic As by methylation that is readily excreted in urine. Cadmium in air has low volatility. Absorption of Cd in human body influences by solubility of Cd compound. Both liver and kidney act to store Cd after oral exposure. When Cd enters body, it will be transported through blood plasma. Cadmium ions bind with anionic groups which primarily bind to metallothionein and albumin. After the binding, it will be filtered in kidney through glomerulus into primary urine and reabsorbed in proximal tubular cells. If metallothionein exceeded, damage to proximal tubular cells occurs [4].

Ministry of Health Malaysia (MOH) [5] reported that As and Cd levels exceeding benchmark were recorded at ex-mining, municipal water supply, solid waste landfill and agricultural areas. In Malaysia, there are several ways of supplying portable water to community, namely, through treated piped water supplies, well water system, gravity feed system (GFS), and piped water and rain water system. The objectives of this study were to determine and compare health implications of consuming As and Cd concentrations in contaminated water supply between urban and rural communities from Langat and Sempeneh rivers.

## 2. Materials and Methods

This cross-sectional study was carried out at 2 areas, namely, Cheras Batu 9 in Hulu Langat District, Selangor, and Batu Kurau in Larut, Matang and Selama (LMS) Dictrict, Perak. Cheras Batu 9 in Hulu Langat District was selected in this study as urban households from this area obtained treated piped water supply from the Langat River Water Treatment Plant. On the other hand, Batu Kurau was selected as the rural community because households in this area obtained water from the gravity feed system (GFS) with water supplied by the Sempeneh River.

The sample size calculated was 50 for each study location: Cheras and Batu Kurau [6]. The study samples were selected from the list of households provided by the village and community head. The respondents were made up of the head of the households or wives who fulfilled the study criteria in term of their age range of 20–50 years, have resided in that area for at least 5 years, with the assumption that they consumed the water supply provided in the area, and that they are healthy and without serious chronic diseases such as diabetes, kidney disease, and hypertension. They should also be free from consuming any long term medication or chemotherapy, smoking, using water filter in the house, and being exposed to products such as As or Cd containing pesticides or paints.

Questionnaire interviews were carried out face to face with respondents to obtain their background information. The questionnaire consists of sections on socioeconomic, demographic, health status, water supply as well as on their occupations which might expose them to As or Cd. Water samples were collected from ten different sampling points including tap water and surface water in Batu Kurau and Cheras areas in order to determine the As and Cd levels in the water supply. Water from the treatment plant and households tap water samples was collected. The tap water was sampled after the tap was turned on to a steady stream and ran for at least 2-3 minutes to remove any stagnant water in the pipeline. For Batu Kurau, water samples were collected from Sempeneh River surface flowing water and from the tap. The respondents' venous bloods were collected by registered medical personnel. Midstream urine samples were collected in the morning from every respondent. Graphite furnace atomic absorption spectrometer (Perkin-Elmer AA600) was used to determine the blood As and urinary Cd among respondents. This instrument uses halo cathode lamp. As levels in blood were detected at 193.7 nm [7], while urinary Cd was detected at 228.8 nm.

Comet assay, known as single cell gel electrophoresis, was used to measure the frequency of DNA damage in the cells. The principle is based upon the ability of denatured, cleaved DNA fragments to migrate out of the cell under the influence of an electric field. The assessment for the DNA damage was based on the DNA “comet” tail shape and the migration pattern of the DNA. The tail length was calculated using the image analysis software (TriTek CometScoreTM).

The urinary NAG levels were analyzed using NAG ELISA Kit and urinary creatinine was analyzed using Reflotron Creatinine. Single cell gel electrophoresis or comet assay is a versatile, sensitive yet simple and economical technique used to measure DNA damage. Comet assay was used to measure frequency of DNA damage in cells. Comet assay was analyzed using steps described by Singh et al. [8] by lymphocytes isolation and centrifuge at 3000 rpm for 5 minutes. The extent of DNA damage was visualized under epiflourescent microscope. Quantitative and statistical data were generated by analyzing the image results using TriTek CometScore freeware V1.5.

Statistical Package for Social Science (SPSS) Version 19.0 was used to analyze all the data. The descriptive test was used to calculate mean, median, mode, and standard deviation. The normality test was used to obtain further information to achieve study objectives. The first and second objectives were analysed using descriptive statistics to determine and analyse the distribution of the Cd, As and urinary NAG levels as well as the genotoxicity impairment of the respondents. Pearson correlation was used when the normality test on both genotoxicity impairment and urinary NAG levels were normal to determine the correlations between two variables. Nonparametric test (Mann-Whitney *U* test) was used for association because urinary NAG levels between two communities were not normally distributed. Multiple linear regression tests were used to analyze the selected variables that influenced the genotoxicity impairment and urinary NAG levels of the study respondents.

## 3. Results

The response rate was 100% in the Batu Kurau rural areas and 90% in Cheras urban areas. There was no significant difference between age, gender, and health between the two communities representing rural and urban. However, years of education, income, and years of residence showed significant difference between the rural and urban communities. The urban respondents were better educated and therefore have better jobs and income. The residence times for the urban respondents were also significantly shorter as they were migrated to the urban areas from other rural and suburban areas looking for better jobs and income.

Table 1 shows that the blood As, urinary Cd, DNA damage, and water As and Cd were significantly (*P* < 0.001) higher in the rural community.  Table 2 shows significant correlations between blood As and DNA damage with household income, years of residence, and total glasses of daily water consumption among the respondents.

Regression analysis in Table 3 shows that the years of residence and seafood intake were significantly related to urinary Cd among respondents.  Table 4 shows that the only variable that was significantly related to the urinary NAG was the urinary Cd (*P* < 0.001).

## 4. Discussion

Since the GFS water supply in the rural Batu Kurau community did not have any treatment, the As and Cd levels were significantly higher than urban Cheras community. In addition, the GFS was built in 1987, uphill, where there was no human activity. However, today, there were human activities such as farming, tree cutting, and housing in the uphill areas.

### 4.1. Urinary Cd

The mean for urinary Cd between study respondents showed the sign of increasing body burden resulting from an environmental exposure. The urinary Cd levels were higher among Batu Kurau community which may be due to the contaminated drinking water consumed every day. The GFS water was directly supplied to the villagers without any treatment. Besides, the Cheras respondents also had low urinary Cd which means that they had the risk of increasing body burden of Cd. Although this community consumed treated water however, it did not ensure safe and clean water because Langat River is currently the most polluted river in Malaysia.

### 4.2. Lymphocyte DNA Damage

The cells with a tail length of below 16.67 *μ*m were classified as “undamaged” and those above 16.67 *μ*m were considered as “damaged”. Since the Cheras respondents had a mean tail length of 11.46 *μ*m, the lymphocytes were undamaged. On the other hand, the Batu Kurau communities with the mean of DNA damage tail length of 21.03 *μ*m showed that their lymphocytes were generally damaged.

### 4.3. Urinary NAG

The increase of NAG isoenzyme seen in the group with low excretion of Cd in urine may possibly be related to programmed cell death induced by Cd exposure. It could be expected that necrotic cell death is the main event with exposure to high levels of Cd. Besides, metallothionein could be one of the factors involved in the induction of apoptosis when tubular cells are exposed to Cd at low levels. It may be due to the presence of insufficient metallothionein; necrosis becomes the main form of tubular cell death when such cells are exposed to high levels of Cd [9].

### 4.4. Selected Variables Which Influenced the Urinary Cd among Respondents

Cd in blood is first bound to albumin and this form is taken up by liver. In the liver, Cd induces the synthesis of metallothionein and binds with it [10]. Thus, the chronic exposure gave rise to renal tubular dysfunction which causes the increase of NAG excretion in urine. The urinary NAG is the biomarker of renal tubular dysfunction caused by Cd exposure. For early kidney biomarkers of Cd exposure, NAG can be detected at levels below 2.0 *μ*g/g creatinine [9]. An increased excretion of urinary NAG resulted from the increasing Cd body burden. Once the Cd is absorbed, it accumulates throughout the lifetime and renal dysfunction may develop if a critical concentration is reached in renal tissue [11].

The years of residence represented the duration of water consumption among respondents and it also influenced the urinary Cd. Most of study respondents were exposed to low levels of Cd for more than 10 years period of time. With low levels exposure to Cd, 30–50% of the body burden of Cd was found in the kidney [11]. The Cd accumulated in kidney, thus it influenced the excretion of urinary Cd. This is supported by past study which showed the urinary Cd levels were higher among the respondents who were born and live in the Cd-contaminated areas due to a long period of exposure [2].

There also was a significant direct relationship between urinary Cd levels and seafood intake (per week) among all respondents. High intake of seafood could increase the absorption of Cd in the body and it would influence the increase of the urinary Cd among respondents. Noonan et al. [10] found majority of urinary Cd values which exceed 2.0 *μ*g/g creatinine demonstrated high exposure and accumulation in body via their diet. Cd absorption after dietary exposure in humans is relatively low (3–5%) but Cd is efficiently retained in the kidney and liver in the human body, with a very long biological half-life ranging from 10 to 30 years [12].

### 4.5. Selected Variables Which Influenced the DNA Damage among Respondents

Blood As showed highly significant correlation with the tail length of DNA damage, which means that blood As would increase DNA damage among the respondents. In this study, the longer their residence in the area, the higher were the As blood concentrations. When the accumulation of As was high, this would lead to the disruption of cell metabolism and retention of DNA damage. When the DNA damage is retained, the mechanism of DNA repair could not cope with the damage anymore. As a result, the cells would die and it would lead to tumor formation. Studies in Taiwan and Bangladesh found that the exposure on the basis of residence contributed to arseniasis being an endemic [13].

Total glass of water consumption which assumed as the total consumption of the water supply in respondents' houses also did not have significant correlation with DNA damage. Besides these, the household income has inverse significant correlation with the DNA damage. With low household income, family members tend to be nutritionally deficient. Deficient nutritional status would reduce the body immunity system by which the DNA can easily be damaged which will most likely lead to cancer development. There are several possible mechanistic explanations for decreases in gene expression observed in the current study. Exposure to As and Cd modifies a variety of transcription factors and signal transduction pathways that are highly dependent on the dose and time course of exposure. Although, it is unclear, however, whether transcription factors actually bind to these sites and elicit biologically functional changes in gene expression. However, the association between As and Cd exposure with decreased gene expression of genes involved in the nucleotide excision repair. Inhibition of nucleotide excision repair capacity by Cd and As may also contribute to its carcinogenic activity [14].

### 4.6. Study Limitations

Environmental factors such as dietary intake of certain foods that might contain Cd and As were not measured as these may be the potential source of these metals to the respondents. Moreover, dietary intake of Cd was not calculated thus Cd intake from these foods were unknown. There were also renal biomarkers for Cd exposure such as urinary *α*1-microglobulin, *β*2-microglobulin, and retinol binding protein (RBP) which were not used due to time constraint and financial limitation, considering that urinary NAG was the most sensitive. There was also no value for normal level of urinary NAG available in literatures to relate it to the degree of renal tubular dysfunction.

## 5. Conclusion

The rural respondents experienced significantly higher lymphocyte DNA damage and higher blood As concentrations which were significantly influenced by their years of residence in the Batu Kurau rural areas. At the same time, they were also exposed to Cd through the use of potable water in which the urinary Cd was significantly higher than Cheras urban respondents and had significant relationship with urinary NAG.

## Figures and Tables

**Table 1:** Comparison of water As and Cd levels, urinary NAG, blood As, urinary Cd, and tail length DNA damage between the two communities (*N* = 100).

	Cheras (*n* = 50)	Batu Kurau (*n* = 50)
	Median (IQR)	Median (IQR)
As in water (mg/L)^**^	0.05 (0.00)	0.18 (0.06)
Cd in water (mg/L)^**^	0.04 (0.01)	0.17 (0.12)
Urinary NAG (U/g creatinine)	0.30 (0.30)	0.30 (0.39)

	Mean (SD)^a^	Mean (SD)^a^

Blood As (*µ*g/dL)^***^	0.90 (0.20)	1.3 (0.20)
Urinary Cd (*µ*g/g creatinine)^***^	1.5 (1.2)	2.6 (1.8)
Tail length of DNA damage (*µ*m)^***^	11 (7.3)	21 (8.9)

^a^Independent *t*-test.

^**^Significant at *P* < 0.01.

^***^Significant at *P* < 0.001.

IQR = inter quartile range.

**Table 2:** Correlation of DNA damage (*µ*m) and selected variables among respondents (*N* = 100).

Variables	Tail length of DNA damage (*µ*m)		
	All respondents	Cheras (*n* = 50)	Batu Kurau (*n* = 50)
	*r*	*r*	*r*
Blood As (*µ*g/dL)	0.40^***^	−0.17	0.17
Urinary Cd (*µ*g/g creatinine)	0.13	−0.03	0.02
Urinary NAG levels (U/g creatinine)	0.04	0.01	−0.00
Age (years)	0.00	0.06	−0.05
Household income (Ringgit Malaysia)	−0.25^*^	0.14	0.12
Residence (years)	0.41^***^	0.07	0.99^**^
Water consumption (glass/day)	0.27^**^	0.11	−0.02

^*^Significant at *P* < 0.05.

^**^Significant at *P* < 0.01.

^***^Significant at *P* < 0.001.

**Table 3:** Relationship between selected variables and urinary Cd levels (*µ*g/g creatinine) among respondents.

Variables^a^	Coefficient regression (*β*)	*t*	*P*
Constant		0.98	0.33
Years of residence	0.25	2.6	0.01^*^
Milk powder (glass/week)	−0.18	−1.9	0.06
Seafood intake (per week)	0.21	2.3	0.03^*^

^*^Significant at *P* < 0.05.

^a^Regression method = stepwise, *F* = 6.194, *R* ^2^ = 0.162.

**Table 4:** Relationship between selected variables and urinary NAG levels (U/g creatinine) among respondents.

Variables	Coefficient regression (*β*)	*t*	*P*
Constant		4.2	>0.001
Urinary Cd level (*µ*g/g creatinine)	0.35	3.7	<0.001^***^

^***^Significant at *P* < 0.001.

Regression method = stepwise, *F* = 13.345, *R* ^2^ = 0.120.
